# Wound Healing and the Use of Medicinal Plants

**DOI:** 10.1155/2019/2684108

**Published:** 2019-09-22

**Authors:** Aleksandra Shedoeva, David Leavesley, Zee Upton, Chen Fan

**Affiliations:** ^1^Skin Research Institute of Singapore, Agency for Science, Technology and Research, A∗STAR, 11 Mandalay Road, Singapore 308232; ^2^Institute of Medical Biology, A∗STAR, Singapore

## Abstract

Cutaneous wound healing is the process by which skin repairs itself. It is generally accepted that cutaneous wound healing can be divided into 4 phases: haemostasis, inflammation, proliferation, and remodelling. In humans, keratinocytes re-form a functional epidermis (reepithelialization) as rapidly as possible, closing the wound and reestablishing tissue homeostasis. Dermal fibroblasts migrate into the wound bed and proliferate, creating “granulation tissue” rich in extracellular matrix proteins and supporting the growth of new blood vessels. Ultimately, this is remodelled over an extended period, returning the injured tissue to a state similar to that before injury. Dysregulation in any phase of the wound healing cascade delays healing and may result in various skin pathologies, including nonhealing, or chronic ulceration. Indigenous and traditional medicines make extensive use of natural products and derivatives of natural products and provide more than half of all medicines consumed today throughout the world. Recognising the important role traditional medicine continues to play, we have undertaken an extensive survey of literature reporting the use of medical plants and plant-based products for cutaneous wounds. We describe the active ingredients, bioactivities, clinical uses, formulations, methods of preparation, and clinical value of 36 medical plant species. Several species stand out, including *Centella asiatica*, *Curcuma longa,* and *Paeonia suffruticosa*, which are popular wound healing products used by several cultures and ethnic groups. The popularity and evidence of continued use clearly indicates that there are still lessons to be learned from traditional practices. Hidden in the myriad of natural products and derivatives from natural products are undescribed reagents, unexplored combinations, and adjunct compounds that could have a place in the contemporary therapeutic inventory.

## 1. Introduction

Our skin is the key to our survival, sensing the environment, maintaining physicochemical and thermal homeostasis, acting as a reservoir of essential nutrients, providing passive and active defence, and responding to trauma and injury [1]. Maintaining these critical functions requires robust and effective mechanisms to protect it from trauma and insult and to repair and replace critical skin functions when damaged or lost. Humans have been treating their wounds for millennia [2]. Traditional wound management is limited by what is immediately at hand or can be acquired locally, such as water, soil, and plant and animal products, and is frequently complemented with ceremony and ritual as an added measure. For millions of people across Asia, Africa, the Middle East, and Latin America, traditional medicines derived from local plants, animals, and natural products are the mainstay of wound care; for some, it is the only source of wound care [3]. We discuss herein some of the evidence supporting the use of medicinal plants as effective and affordable treatments for cutaneous wounds.

## 2. Cutaneous Wound Healing

**Figure 1:**
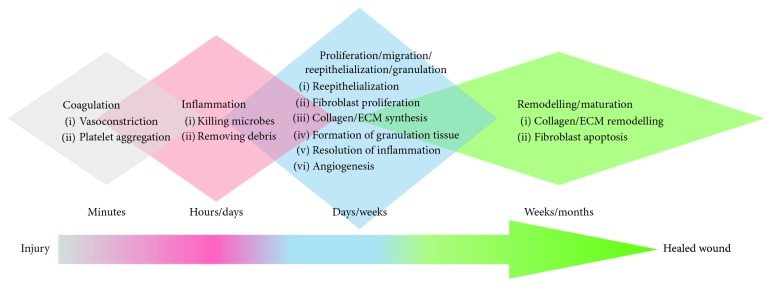
Wound healing cascade—humans. The wound healing process is an orderly sequence of overlapping, interacting processes commonly categorised into four distinct phases: coagulation, inflammation, proliferation/migration/reepithelialization/granulation, and remodelling/maturation. (1) Coagulation: a clot is formed, providing a temporary barrier to fluid loss and pathogen entry, restores haemostasis; acts as a reservoir of bioactive factors and antimicrobials; provides provisional ECM which supports immune cell infiltration and migration; and initiates tissue repair pathways. (2) Inflammation: damage-associated molecular patterns, free radicals, and reactive molecular species are signals to recruit immune cells; increased blood vessel leakiness; release of antimicrobial species; infiltrating immune cells secretes amplifying alarmin (also known as DAMPs) signals; and activation of keratinocytes and fibroblasts. (3) Proliferation/migration/reepithelialization/granulation: migration and proliferation of keratinocytes, fibroblasts, endothelia; resolution of inflammation; collagen/ECM synthesis; decreased vessel permeability; new capillary and lymphatic vessel angiogenesis; reepithelialization; and de novo formation of granulation tissue. (4) Remodelling/maturation: collagen/ECM turnover (synthesis and degradation); ECM reorganisation and realignment; ECM contraction; endothelia and fibroblast apoptosis; repigmentation.

Maintaining homeostasis is critical for the survival of the organism; hence, skin needs and possesses a robust and effective repair mechanism. Cutaneous wound healing is the process by which skin repairs itself following injury caused by surgery, trauma, and burns [4]. The healing process is classically divided into 4 phases (Figure 1): coagulation (a.k.a. haemostasis), inflammation and proliferation (a.k.a. granulation), and remodelling (a.k.a. maturation) [5]. Upon injury, a fibrin clot rapidly forms to restore haemostasis [6, 7]. Platelets present in the blood trigger the clotting cascade and secrete several growth factors, initiating wound healing [8]. In the following inflammation phase, neutrophils migrate into the wound site engulfing foreign debris and killing bacteria by phagocytosis and releasing proteolytic enzymes [8, 9]. Coincidently, blood monocytes infiltrate the injury site and differentiate into macrophages, releasing proteases to debride the wound [8], and secrete a mixture of bioactive molecules, including transforming growth factor-beta 1 (TGF-*β*1), that stimulates the migration of fibroblasts and epithelial cells [10]. The proliferation phase usually starts about 3 days after wounding; it involves diverse activities including angiogenesis (by endothelial cells), granulation tissue formation (by fibroblasts), and reepithelialization (by keratinocytes) [11, 12]. In this stage, fibroblasts produce a large amount of extracellular matrix (ECM), mainly collagen, to form the granulation tissue which replaces the damaged tissue. Meanwhile, the keratinocytes migrate, proliferate, differentiate, and re-form a functional epidermis (reepithelialization), closing the lesion and protecting underlying tissues from further trauma [13]. As the wound matures, the characteristic disorganized ECM of granulation tissue is actively remodelled by the dermal fibroblast cell population [14], whose numbers are progressively reduced through apoptosis [15]. The outcome of wound healing is scar tissue (aka fibrosis) with sparsely distributed fibroblasts within a collagen-rich ECM. Compared to the original tissue, scar tissue, having distinct texture and reduced biomechanical and functional properties, is characteristically altered [16].

**Figure 2:**
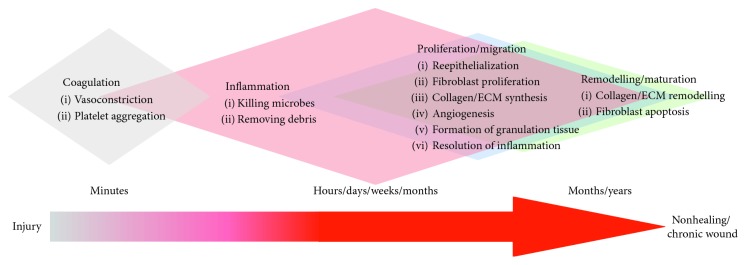
Nonhealing/chronic wounds—humans. The orderly sequence of overlapping, interacting wound healing processes fails to progress in chronic wounds, frequently due to failure to resolve inflammation. (1) Coagulation: usually unaffected. (2) Inflammation: damage-associated molecular patterns, free radicals, and reactive molecular species; high pH; functional activation of proteases, senescence of keratinocytes, and fibroblasts (vessel permeability sustained-aetiology specific). (3) Proliferation/migration: initiation of de novo granulation tissue formation; failure to sustain proliferation; failure to initiate angiogenesis; failure of keratinocytes to migrate and reepithelialise (failure of wound closure); failure to resolve inflammation; and failure to accumulate ECM. (4) Remodelling/maturation: fails to initiate reorganisation and maturation of ECM.

Healing of acute wounds follows an orderly sequence of overlapping, interacting physiological processes (Figure 1). This sequence can take over a few days in juveniles or over a few weeks in adults to occur. Most wounds heal without complication and reestablish homeostasis, skin barrier function, pliability, and physiological functions in less than 4 weeks. Clinical evidence indicates that shorter periods to wound closure are associated with reduced fibrosis and scarring. In contrast, full-thickness wounds and wounds that are slow to heal are associated with increased fibrosis, developing in some individuals into hypertrophic scars and keloids. Deep, full-thickness, and partial-thickness wounds that do not heal within 6 weeks appear to “stall” and fail to progress through the phases of healing described in Figure 1 (Figure 2). These hard-to-heal wounds are considered to be “chronic” wounds [17]. Hard-to-heal wounds become “chronic” for a number of reasons, including underlying conditions such as diabetes, vascular disease, hyperglycaemia, ischemia, and neuropathy. The underlying cause of the wound is often used to describe the wound: diabetic foot ulcers, venous leg ulcers, arterial leg ulcers, and pressure ulcers.

Nonhealing, chronic wounds clearly pose a risk to the health and well-being of the individual; patients often suffer from pain, impaired mobility, excessive exudates, wound malodour, and restricted social life [18], resulting in substantial disruption, morbidity, and indirect costs to social and healthcare systems [19]. As many as 1-2% of individuals in all populations worldwide will acquire a chronic wound during their life-time [20]. In the USA, chronic wounds are reported to affect 6.5 million people and cost over US $25 billion each year [21]. Alarmingly, the burden of chronic wounds is expected to intensify due to global increases in vascular diseases, diabetes, obesity, metabolic syndrome, and the general aging of the population [21]. Although the mechanisms of wound healing are relatively well known, the pathogenesis of chronic wounds remain poorly defined [22]. It is generally accepted that chronic wounds result from some dysregulation of the normal wound healing process. For example, microbial biofilms, overexpression of inflammatory cytokines, high levels of proteases and reactive oxygen species (ROS), and reduced mitogenic activity stall wound healing in the inflammation phase, inhibiting progression to the proliferation and reepithelialization phases. In addition, overactive matrix metalloproteinases (MMPs) have been shown to contribute to delayed healing [23]. The result is a wound that remains open, does not heal, and becomes chronic [24].

Selecting an appropriate clinical strategy to manage cutaneous wounds is dictated by the aetiology underlying each wound. Consideration is given to (1) removing nonvital (necrotic) tissue, termed debridement; (2) inflammation or infection; (3) controlling moisture (too wet or too dry); and (4) state of the tissue surrounding the wound [25]. This approach has its roots in Greek and Roman medicine [26], where removing these “barriers to healing” was prescribed to allow the healing cascade to progress to completion. Debridement is considered to benefit wounds, restarting the healing process by returning it to an acute presentation. Debridement exposes healthy and well-perfused tissue, facilitating cell proliferation and migration [27]. In addition to removing dead and necrotic tissue, debridement effectively reduces, if not removes, proinflammatory factors, damage-associated molecular patterns (DAMPs), and pathogen-associated molecular patterns (PAMPs). Debridement also removes “extracellular traps” and microorganisms from the wound. Microorganisms in wounds have long been considered deleterious [28]; however, recent evidence suggests that not all microbes impede healing. Microbial pathogens such as *Staphylococcus* sp., *Streptococcus* sp., *Propionibacterium* sp., and *Pseudomonas* sp. are commonly equated with infection, while others including *Malassezia* sp., *Candida* sp., and *Corynebacterium* sp. can be isolated from noninfected wounds and may even contribute to wound healing [29]. The optimal frequency and when to perform debridement, however, remains unclear [27]. Dressings provide a physical barrier to reinfection from commensal and adventitious microorganisms, can deliver antimicrobial agents (e.g., honey and iodine), and, in some designs, absorb wound exudates, providing a measure of moisture control.

## 3. Traditional Medical Practices

Traditional medicine is often described by practitioners of “modern” (western) medicine using sceptical terms such as “alternative,” “nonconventional,” “indigenous,” and “complementary,” when in fact many of the techniques and practices of “modern” medicine are little different from traditional practices when it comes to wounds. Traditional approaches depend almost entirely upon natural resources, such as water, plants, animals, and minerals, and continue to be valued and widely practiced by a majority of the world's population [3]. The practice of traditional Chinese medicine (TCM) is based on the Five-Phases theory and Yin-Yang theory, recorded in the ancient Chinese medical documents such as “*Shen Nong Ben Cao Jing*” and “*Ben Cao Gang Mu.*” Many, but not all, TCM makes extensive use of plants, ensuring it is effective, affordable, and accessible [30]. Interestingly, of the new anticancer drugs developed between 1940 and 2002, approximately 54% were derived from natural products [31]. Another study has determined that of all current pharmaceutical products, about 73%, include ingredients derived from natural products [32]. The therapeutic activity of many traditional medicines are conferred by natural ingredients produced within the plant; consequently, the efficiency of TCM preparations can vary widely and are determined by the genotype, environmental, and growing conditions encountered by each source plant [30, 33]. Urbanization and industrialization of pharmaceutical engineering have increased demand for “off the shelf” TCM products with consistent composition, quality, and clinical efficacy. Concomitantly, industrialization has also introduced rigorous product testing for evidence of biological activity and clinical efficacy.

## 4. Traditional Use of Medical Plants in Wound Healing

### 4.1. *Aloe vera*

Applied to wounds for over 5000 years by Egyptians, Romans, indigenous peoples of Africa Asia, and the Americas, *Aloe vera* continues to be a first-line treatment for burns, ulcers, and surgical wounds [34]. *Aloe vera* contains many natural bioactive compounds, including pyrocatechol, saponins, acemannan, anthraquinones, glycosides, oleic acid, phytol, as well as simple and complex water-soluble polysaccharides [35]. Acetone extracts from the leaves of *Aloe vera* exhibit stronger antimicrobial activity than alcohol and aqueous extracts. Gram-positive bacterial species appear to be more sensitive than Gram-negative species to *Aloe vera* [36]. Compounds with known antimicrobial activity are saponins, acemannan, and anthraquinone derivatives [37].

Acemannan, a major mucopolysaccharide (mesoglycan) from *Aloe vera*, is a potent stimulator of macrophage and T-cell activity and induces the transcription of proinflammatory mRNAs (including IL-1*α*, IL-1*β*, IL-6, TNF-*α*, PGE2, and nitrous oxide) [38]. Mesoglycan moieties bind and capture endogenous mitogen inhibitors and reactive oxygen species and promote phagocytosis. Coincidently, glycans stabilize secreted cytokines, growth factors, and other bioactives, prolonging their activity. Topically applied acemannan has been reported to significantly reduce the time to wound closure in a rat wound healing model, acting via cyclin D1 and AKT/mTOR signal pathways [39]. *Aloe vera* glycans are also reported to significantly improve de novo formation of granulation tissue by an unknown mechanism [40].

### 4.2. *Arctium lappa*

*Arctium lappa*, commonly known as burdock, is a widely cultivated perennial herb [41]. *Arctium lappa* is used in North America, Europe, and Asia to treat sore throat and skin pathologies such as boils, rashes, and acne [42, 43]. Scientific analyses demonstrate *Arctium lappa* has antioxidant [44], anti-inflammatory [45], antidiabetic [46], antimicrobial [47], antiviral [48], anticancer [49], and hepatoprotective [50] properties. The root extract of *Arctium lappa* has been shown to significantly improve dermal ECM metabolism, affecting glycosaminoglycan turnover and reducing visible wrinkles in human skin *in vivo* [51]. *Arctium lappa* is also reported to regulate cell adhesion and gene expression in canine dermal fibroblasts, affecting the Wnt/*β*-catenin signalling pathway, known to be a key regulator of wound healing [52]. In a pilot study of one commercial preparation including *Arctium lappa*, Burns and Wounds™ topical ointment (B&W), pain and healing of first- and second-degree burns in humans was demonstrated to be managed more effectively than the control treatment [53].

### 4.3. *Astragalus propinquus* and *Rehmannia glutinosa*

The root of *Astragalus propinquus* is a common TCM for the treatment of urinary retention and oedema [54]. The root of *Rehmannia glutinosa* has been broadly used in hemorheology and diabetes-related diseases [55]. A formulation combining the root of *Astragalus propinquus* and *Rehmannia glutinosa* was initially reported to be clinically effective for the treatment of diabetic foot ulcers [56]. This outcome has subsequently been corroborated in diabetic rats [57]. Tam et al. reported that the root of *Astragalus propinquus* and *Rehmannia glutinosa* promote diabetic wound healing and postischemic neovascularization by improving angiogenesis and attenuating tissue oxidative stress in diabetic rats [58]. Zhang et al. demonstrated that the root of *Astragalus propinquus* and *Rehmannia glutinosa* activate the TGF-*β*1 signalling pathway and stimulate increased deposition of ECM in human skin fibroblasts [59].

### 4.4. *Ampelopsis japonica*

Growing throughout eastern Asia and eastern North America, the roots of *Ampelopsis japonica* are used as a traditional treatment for burns and ulcers, amongst other indications [60]. Multiple pharmacological activities have been documented for *Ampelopsis japonica*, including neuroprotective [61], antimicrobial, and anticancer [62] activities. Lee et al. demonstrated that ethanol extracts from dried roots of *Ampelopsis japonica* accelerated the healing of cutaneous scald injury in rats [63]. Tumour necrosis factor-alpha (TNF-*α*) and TGF-*β*1 were observed to be elevated 2 days after injury and declined as healing progressed. In contrast, interleukin-10 (IL-10) was found to be elevated after 14 days, coincident with wound closure [63]. When compared with wounds treated with Vaseline® (petroleum jelly) or silver sulfadiazine, topical treatment with ethanolic *Ampelopsis japonica* improved reepithelization, granulation tissue formation, vascularization, and collagen deposition [63].

### 4.5. *Andrographis paniculata*

*Andrographis paniculata*, also known as green chiretta, is used in China, India, and south east Asian countries as a traditional treatment for fever, snake bite, dysentery, infections, wounds, and itchiness [64–67]. Extracts from *Andrographis paniculata* exhibit antioxidant [68], anti-inflammatory [69], antidiabetic [70], anticancer [66], antimicrobial [71], antiviral [72], antimalarial [73], hypotensive [74], immunostimulatory [66], and hepatoprotective [75] activities. In one study, wound closure in rats was observed to be significantly enhanced after treatment with a 10% aqueous leaf extract of *Andrographis paniculata* [76]. Animals treated with *Andrographis paniculata* exhibited reduced inflammation, reduced scarring, increased angiogenesis, and an increased number of collagen fibres in healed wounds [76]. Andrographolide, a bicyclic diterpenoid isolated from the leaves of *Andrographis paniculata*, has been formally evaluated in clinical trials and shown to have positive effects on several autoimmune disorders [77].

### 4.6. *Angelica sinensis*

The dried root of *Angelica sinensis* is widely used in TCM prescriptions for the management of female maladies, inflammation, headaches, mild anemia, fatigue, and hypertension [78]. *Angelica sinensis* possesses pharmacological activities including anti-inflammatory [79], anticancer [80], antioxidant effects [81], and immune modulator [82]. Extracts from *Angelica sinensis* have been shown to activate an antiapoptotic pathway and enhance cell proliferation, collagen secretion, and cell mobility in human skin fibroblasts [83]. Extracts have also been shown to stimulate glycolysis and calcium fluxes, increasing cell viability during tissue repair [83]. The role of *Angelica sinensis* in angiogenesis remains unclear, with several studies reporting contradictory effects of *Angelica sinensis* on de novo blood vessel growth. An aqueous extract of *Angelica sinensis* was reported to promote blood vessel growth via activation of JNK1/2 and p38 phosphorylation, resulting in enhanced VEGF expression [84, 85]. In contrast, *n*-butylidenephthalide, a bioactive isolated from *Angelica sinensis*, inhibits cell cycle progression, induces apoptosis, and attenuates angiogenesis [86].

### 4.7. *Blumea balsamifera*

Endemic throughout the tropics and subtropics of Asia, *Blumea balsamifera* (also known as ngai camphor) is used widely as a traditional medicine. In the Philippines, *Blumea balsamifera* is known as sambong and is used as a diuretic. In Ayurveda, *Blumea balsamifera* is known as kakoranda and is used to treat fevers, coughs, aches, and rheumatism. Leaf extracts are directly applied to treat eczema, dermatitis, skin injury, bruises, beriberi, lumbago, menorrhagia, rheumatism, and skin injury [87]. Extracts from *Blumea balsamifera* demonstrate a variety of bioactivities; including antimalarial [88], antitumour [89], antifungal [90], and antiobesity [91] properties. Pang et al. reported that oils from *Blumea balsamifera* improve wound healing in mice by promoting angiogenesis, perfusion, collagen deposition, formation of organised granulation tissue, reepithelialization, and wound closure [92].

### 4.8. *Boswellia sacra*

Frankincense, a resinous extract from *Boswellia sacra*, is valued in Africa, India, and the Middle East for the treatment of trauma and inflammatory diseases such as rheumatoid arthritis [93, 94]. It has also been reported that the boswellic acid acetate extracted from frankincense induces apoptosis and differentiation in melanoma and fibrosarcoma cells [95]. It is a key component of ANBP, a TCM consisting of pulverised *Agrimonia eupatoria* (A), *Nelumbo nucifera* (N), *Boswellia sacra* (B), and pollen from *Typha angustifoliae* (P). ANBP stimulates Smad-dependent pathways in the TGF-*β*1 signalling cascade [96]. Using a rabbit ear model of hypertrophic scarring, Hou et al. demonstrated that ANBP moderates inflammation and accelerates the growth of organized granulation tissue and reepithelialization, events that reduce scar formation [96]. Intriguingly, ANBP was also noted to attenuate collagen biosynthesis and accelerate the maturation of the collagen extracellular matrix, contributing to reduced scarring and improved skin tissue repair. Recently, Hou et al. further demonstrated that ANBP reduced the time of wound closure in diabetic mice via direct effects on neovascularization [97].

### 4.9. *Caesalpinia sappan*

The heartwood of *Caesalpinia sappan* is well known for its qualities as a dye and has been used in TCM to improve blood circulation and reduce oedema and pain [98]. Homoisoflavonoids isolated from *Caesalpinia sappan* have been found to possess antiallergic [99] and anti-inflammatory [100] attributes and to inhibit viral neuraminidase activity [101]. Ethanol extracts of *Caesalpinia sappan* exhibit effective antibacterial activity against *Staphylococcus aureus*, methicillin-resistant *Staphylococcus aureus* (MRSA), *Pseudomonas aeruginosa*, *Acinetobacter baumannii*, *Escherichia coli,* and *Klebsiella pneumoniae* [102]. Unexpectedly, the ethanol root extract from *Caesalpinia sappan* also stimulates dermal fibroblast proliferation, migration, and collagen synthesis [103], in turn improving cutaneous wound healing.

### 4.10. *Calendula officinalis*

*Calendula officinalis*, commonly known as pot marigold, is a very widely distributed plant used for the treatment of a variety of skin conditions, such as wounds, burns, and dermatitis [104, 105]. A range of pharmacological activities are ascribed to *Calendula officinalis*, including anti-inflammatory, antioxidant, antibacterial, antiviral, antifungal, and anticancer activities [106]. However, the exact mechanisms involved in its activities on the wound healing remain unknown. Studies using cultures of human and murine fibroblasts demonstrated that extracts of *Calendula officinalis* stimulate fibroblast migration and proliferation in a PI3K-dependent manner [107, 108]. Extracts from the flower of *Calendula officinalis* stimulate granulation tissue formation by altering the expression of connective tissue growth factor (CTGF) and *α*-smooth muscle actin (*α*-SMA) in excisional wounds of BALB/c mice *in vivo* [109]. *Calendula officinalis* is also reported to enhance angiogenesis *in vivo*, demonstrated using the chicken chorioallantoic membrane (CAM) assay and a cutaneous wound healing model in rats [110].

### 4.11. *Camellia sinensis*

Green tea, an aqueous extract made from the leaves of *Camellia sinensis*, is revered throughout Asia for its reputed health benefits [111]. Centuries of anecdotal evidence has been experimentally validated by demonstrating that *Camellia sinensis* has antioxidant [112], anti-inflammatory [113], antimicrobial [114], anticarcinogenic [115], antiaging [116], antiobesity [117, 118], cardioprotective [119], and neuroprotective [120] activities. Catechins, the polyphenolic compounds from *Camellia sinensis*, are primarily responsible for these pharmacological activities [121]. The major catechin, (-)-epigallocatechin-3-gallate (EGCG) [111], stimulates the proliferation and differentiation of keratinocytes [122]. Klass et al. found that EGCG suppresses TGF-*β* receptors by modifying TGF-*β* signalling, reducing MMP-1 and MMP-2 expression, and attenuating synthesis of collagen type 1 in human dermal fibroblasts. These properties suggest that EGCG is a potential antiscarring agent [123]. In addition, EGCG was demonstrated to induce keloid shrinkage [124] and inhibit growth and pathological features of keloids by suppressing STAT3 signalling [125]. Methanol extracts from *Camellia sinensis* reportedly increase fibroblast proliferation and collagen synthesis [115]. Furthermore, *in vivo* studies have demonstrated that *Camellia sinensis* significantly improves wound healing by increasing angiogenesis in rats [121, 126]. Extracts from *Camellia sinensis* are also reported to improve wound healing in a diabetic mouse model [127].

### 4.12. *Carthamus tinctorius*

Seeds from *Carthamus tinctorius*, or safflower, are a popular source for cooking oil in many countries. Less widely known, *Carthamus tinctorius* also has a long history as an ingredient in TCM formulations for the treatment of blood disorders. Recent experimentation has identified it is associated with a wide range of biological activities, including vasodilation, immune modulation, anticoagulation and thromboprophylaxis, antioxidation, antihypoxic, antiaging, antifatigue, anti-inflammation, antihepatic fibrosis, anticancer, and analgesia [128]. Interestingly, safflower seed oil has also been shown to inhibit melanogenesis in B16 melanoma cells, making it a promising candidate for skin whitening [129]. Hydroxysafflor yellow A (HSYA), the major water-soluble monomer of safflower yellow pigments, has been shown to protect against cerebral and myocardial ischemia [130], conferring antioxidant [131], anti-inflammatory [132], proangiogenic [133], and apoptosis-inhibiting [134] properties. Topical application of HSYA at low dose (4 mg/mL) improves diabetic wound healing, promoting neovascularization, reepithelialization, and granulation tissue formation in streptozotocin-induced diabetic rats [130]. In contrast, at high doses (≥10 mg/mL), wound healing is inhibited [135, 136].

### 4.13. *Celosia argentea*

*Celosia argentea*, also known as silver cock's comb, is used in traditional medicine to treat skin sores, eruptions, ulcers, mouth ulcers, and other skin diseases [137]. Leaf extracts of this plant possess antioxidant [138], hepatoprotective [139], antidiabetic [140], and antimicrobial [141] activities. Priya et al. demonstrated that an alcohol extract of *Celosia argentea* accelerates burn wound closure in rats by increasing collagen and hexosamine content in granulation tissue wounds. In addition, the extract increased the proliferation and motility of primary rat dermal fibroblasts [137].

### 4.14. *Centella asiatica*

*Centella asiatica*, also known as Asiatic pennywort, has been used to promote wound healing for eons [142]. Extracts from the aerial parts of *Centella asiatica* are reported to improve the healing of chronic ulcers in Sprague-Dawley rats in terms of width, depth, and length [142]. Wounds associated with acute radiation dermatitis in rats were observed to heal earlier when treated with extracts from *Centella asiatica* compared to the no-treatment control group [143]. Asiaticoside isolated from *Centella asiatica* has been found to enhance collagen deposition and epithelialization in a punch wound model in the guinea pig [144]. Triterpenes isolated from *Centella asiatica* elevate collagen remodelling and glycosaminoglycan synthesis in a rat wound model [145]. Furthermore, oral administration of madecassoside from *Centella asiatica* was shown to facilitate collagen synthesis and angiogenesis in a mouse wound model [146].

### 4.15. *Cinnamomum cassia*

*Cinnamomum cassia* is a commonly used spice and flavouring agent, and the bark of *Cinnamomum cassia* is also used to increase blood circulation and as an analgesic [147]. *Cinnamomum cassia* is frequently formulated with other herbs; it is one of the seven botanical components of Shexiang Baoxin pill (SBP), a well-known TCM prescribed for chest pain and discomfort associated with coronary artery disease [148]. SBP is currently the subject of a randomized double-blinded clinical trial for the treatment of coronary artery disease not amenable to revascularization [149]. Attention is also focussed on SBP anti-inflammatory [150] and anticancer activities [151, 152], as well as its impact on hypertension, insulin resistance, and noninsulin-dependent diabetes mellitus [153]. *In vitro* and *in vivo* studies indicate that cinnamaldehyde, a bioactive component from *Cinnamomum cassia*, is a natural insecticide, is an antimicrobial, antidiabetic, antilipidemic, anti-inflammatory, and neuroprotective agent [154], and activates PI3K/AKT and MAPK signalling pathways, increasing VEGF expression, and stimulating angiogenesis in human umbilical vein endothelial cells [147]. Cinnamaldehyde is also reported to improve wound healing in zebrafish [147].

### 4.16. *Commiphora myrrha*

Myrrh, the resinous exudate produced by *Commiphora myrrha* [155], has well-documented antioxidant [156], anti-inflammatory [157], antibacterial [158], and analgesic [159] activities. Medicinal applications of myrrh include the treatment of gastrointestinal diseases, fractures, arthritis, obesity, parasitic infections, and as an anticoagulant [160–162]. Myrrh has been used topically to clean wounds, reduce oedema, and provide pain relief (analgesia) [163]. Myrrh is commonly used in combination with other ingredients. Galehdari et al. showed that the combination of myrrh, *Adiantum capillus-veneris*, *Aloe vera,* and *Lawsonia inermis*, significantly improved wound healing in diabetic mice [164]. The short-term application of myrrh effectively reduces pain and controls the recurrence of mouth ulcers in humans [165]. In common with several other herbal preparations described here, myrrh is found to modify the expression of TGF-*β*1 and VEGF in mouse dermal fibroblasts *in vitro*, suggesting a common mechanism of action [166].

### 4.17. *Curcuma longa*

Curcumin, an active substance found in the root of *Curcuma longa* and a member of the ginger family, has long been used as a medicine and as food seasoning [167]. Practitioners of traditional Ayurveda medicine use curcumin to treat inflammation, respiratory disorders, liver disorders, and diabetes [168]. In traditional Chinese medicine, curcumin is a favoured treatment for abdominal pain. Having widespread use for centuries by diverse ethnic groups, curcumin is one of the most extensively studied nutraceuticals. This highly pleiotropic molecule has been demonstrated to interact with key cellular pathways at transcription, translation, and posttranslational levels. Target pathways include proinflammatory cytokines, apoptosis, NF–κB, cyclooxygenase-2, 5-LOX, STAT3, C-reactive protein, prostaglandin E2, prostate-specific antigen, cell adhesion molecules, phosphorylase kinase, transforming growth factor-*β*, triglycerides, ET-1, creatinine, heme oxygenase-1, AST, and ALT [169]. The subject of more than 100 clinical trials, *in vivo* studies, have largely focused on curcumin as a treatment for epithelial cancers. Experimental findings from these *in vivo* studies and *in vitro* experiments indicate curcumin elicits most of its beneficial effects via altering the pericellular and extracellular matrix [168]. Perhaps, it is therefore not unexpected that curcumin enhances fibroblast proliferation, granulation tissue formation, and collagen deposition in cutaneous wound healing [170].

### 4.18. *Daphne genkwa*

*Daphne genkwa*, one of the 50 fundamental herbs used in TCM, grows in the Yellow and Yangtze Rivers regions in China. *Daphne genkwa* is used as an anticonvulsant, analgesic, diuretic, antitussive, expectorant, and mild sedative agent [171–174]. The principal bioactives isolated from *Daphne genkwa* are biflavonoids, coumarin, diterpenes, and triterpenes. These confer anti-inflammatory [175], antitumour [176, 177], immunoregulatory [178, 179], and antimelanogenesis [172] activities. Flavonoids extracted from the flowers of *Daphne genkwa* stimulate the ERK/MEK pathway regulating fibroblast proliferation and the expression of collagen (*COL1A1* and *COL3A1*), resulting in improved wound healing [171].

### 4.19. *Entada phaseoloides*

*Entada phaseoloides*, also known as St. Thomas bean, is a liana in the pea family of climbing vines common throughout lowland tropical forests and coastal forests of Africa, Australia, Asia, and Western Pacific. The bark and seeds of *Entada phaseoloides* are rich in saponins and tannins and are used as analgesic, bacteriocide, haemostatic, and anticancer agents and as a topical treatment for skin lesions [180, 181]. Su et al. reported that extracts enriched with tannins from *Entada phaseoloides* reduced the time taken to heal infected wounds in rats. Analyses of the data concluded that the improved wound healing was due to the antibacterial, proproliferative, and promigration activity of the *Entada phaseoloides* extracts [182]. These data are yet to be validated in human patients.

### 4.20. *Hibiscus rosa-sinensis*

*Hibiscus rosa-sinensis*, or shoeblackplant, is an evergreen shrub native to tropical South Eastern Asia [183]. The flowers of *Hibiscus rosa-sinensis* are edible. Traditional texts describe preparations of the leaves and flowers promote hair growth and prevent greying [184]. Alcoholic extracts of *Hibiscus rosa-sinensis* flowers are claimed to provide women with control of their fertility [185]. Extracts from *Hibiscus rosa-sinensis* have also been found to have antibacterial [186] and wound healing properties [187]. They attenuate inflammation, enhance fibroblast proliferation, and collagen deposition, as well as upregulate VEGF and TGF-*β*1 expression in rat excisional wounds [188].

### 4.21. *Ganoderma lucidum*

*Ganoderma lucidum*, the lingzhi mushroom, is well known to the Chinese, Korean, and Japanese as “the mushroom of immortality” [189, 190]. Used in TCM to boost the patient's immune system [191], *Ganoderma lucidum* stimulates a variety of pharmacobiological responses including immune modulation, inflammation modulation, anti-infective [192–194], antioxidant [195], cardioprotection [196], and antihyperlipidemia [197] activities. Clinical studies suggest that taking *Ganoderma lucidum* daily is beneficial and is reported to reduce the number of tumours in patients with colorectal adenomas; circulating viral particles in patients infected with hepatitis B; and symptoms of hypertension [189, 198–202]. Laboratory-based studies reveal that components from *Ganoderma lucidum* interact with and modulate key enzymes with known roles in lipid metabolism. However, clinical findings remain equivocal and suggest that *Ganoderma lucidum* is most effective when used as an adjunct with other therapies [203]. Polysaccharide extracts from the fruiting body of *Ganoderma lucidum* have been shown to improve wound healing in diabetic rats, potentially by stimulating fibroblast proliferation and migration [190], angiogenesis, and quenching oxidative stress [204]. Nevertheless, these responses may also represent indirect responses to *Ganoderma lucidum* via its established stimulation of humoral immunity.

### 4.22. *Ligusticum striatum*

The rhizome of *Ligusticum striatum* is another one of the 50 fundamental herbs used in TCM. It has a long history of use support cardiovascular and cerebrovascular well-being. It is commonly indicated for the treatment and prevention of ischemic disorders, menstrual disorders, and headache [205–207]. Thus far, about 174 chemical components have been isolated from *Ligusticum striatum*, among which phthalide lactones and alkaloids are the most numerous, pharmacologically active species [207]. It has been reported that essential oils from *Ligusticum striatum* inhibit dermal scarring in the rabbit ear scar model [208].

### 4.23. *Lonicera japonica*

*Lonicera japonica*, also known as honeysuckle, has a notable place in traditional medicine throughout its native range of Japan, Korea, and China, where it has been used for thousands of years to treat infectious diseases [209]. In the 1980s, the Chinese State Ministry of Health performed extensive pharmacological and clinical analyses of *Lonicera japonica* and identified broad-spectrum antimicrobial, anti-inflammatory, antipyretic, antioxidant, anticancer, hepatoprotective, and antihyperlipidemic capabilities [210, 211]. More recently, Chen et al. demonstrated that the ethanol extracts of the flowering aerial parts of *Lonicera japonica* also support reepithelization, angiogenesis, granulation tissue formation, and contraction during cutaneous wound healing [212]. The plant may be consumed as a “health food,” providing some protection from gastric ulceration although, at high doses, it can cause some neurological pathologies [210].

### 4.24. *Paeonia suffruticosa*

*Paeonia suffruticosa*, also known as moutan peony, has been bred for millennia [213]; over 1000 distinct cultivars are now available. The root bark of *Paeonia suffruticosa* is the source for bioactive ingredients used for TCM preparations. Pharmacological investigation of *Paeonia suffruticosa* has demonstrated it has antioxidant [214], neuroprotective [215], antitumour [216], anti-inflammatory [217], and antidiabetic [218] properties. The dried root of *Paeonia suffruticosa* is commonly applied to cracked skin to assist healing and relieve pain [219]. When tested *in vitro* at low concentrations (≤10 *μ*g/mL), *Paeonia suffruticosa* is found to stimulate the viability and proliferation of human primary dermal fibroblasts and HaCaT keratinocytes, suggesting its potential use as a wound healing therapy [220].

### 4.25. *Panax ginseng*

*Panax ginseng* is one of the most popular medicinal plants consumed in China, Japan, Korea, and Eastern Siberia to improve thinking, concentration, and memory. It is also claimed to support immunity and physical stamina and to reduce fatigue [221]. *Panax ginseng* is thus used to treat depression, anxiety, and chronic fatigue syndrome [222]. *Panax ginseng* has been demonstrated to induce vasodilation [223], control blood lipids [224], reduce inflammation [225], and confer antioxidant [226], anticancer [227], antibacterial [228], antiallergic [229], antiaging [230], and immunomodulating [231] activities. *Panax ginseng* contains many bioactive substances, among which a class of saponins (termed ginsenosides by Asian researchers and panaxosides by Russian researchers) represent the most potent active constituents of *Panax ginseng* [232].

The root extracts of *Panax ginseng* have been shown to protect skin in C57BL mice from acute UVB irradiation [233] and significantly improve healing after laser burn injury and excisional wounding [221, 234, 235]. Studies demonstrate *Panax ginseng* extracts enhance keratinocyte migration [221, 236], as well as stimulate proliferation [237] and increase collagen synthesis in human dermal fibroblasts [238] *in vitro*. In addition, Choi demonstrated that the ginsenoside Rb2, isolated from *Panax ginseng*, induces the formation of the epidermis in raft culture via increased expression of epidermal growth factor and its receptor, fibronectin and its receptor, and keratin 5/14 and collagenase I [239], all of which have critical roles in wound healing.

### 4.26. *Panax notoginseng*

*Panax notoginseng*, not to be confused with *Panax ginseng* and other ginsengs, is used to stop bleeding, reduce oedema, reduce bruising, and reduce pain [240, 241]. Terpene saponins isolated from the leaves of *Panax notoginseng* possess substantial pharmacological activities, including antioxidative effects [242], anti-inflammatory effects [243], immunostimulation [244], neuroprotective effects [245], anticancer [246], and antidiabetic activities [247]. Terpene saponins stimulate VEGF expression and angiogenesis, key factors in wound healing [240, 241]. Mechanism of action studies has found *Panax notoginseng* flower extracts block NF-κB signalling [248, 249], thus affecting the expression of inflammatory cytokines, including IL-6, known to contribute to keloid pathogenesis [250, 251].

Interestingly, saponins isolated from *Panax notoginseng* exhibit antihaemostatic (antiplatelet and anticoagulant) activity when assayed *in vitro* and *in vivo* in a rat model [252]. It was proposed that when administered orally, key bioactive constituents responsible for the haemostatic activity could be modified, which does not occur when administered topically. Of particular note, it is now evident that ginsenosides exhibit significant stereospecific differences in pharmacokinetic properties, including absorption, distribution, and metabolism [253]. These findings may account for some of the confusing and contradictory experimental observations. For example, 20(R)-ginsenoside Rh2 inhibits osteoclastgenesis without cytotoxicity. In contrast, 20(S)-ginsenoside Rh2 is strongly cytotoxic for osteoclasts [254]. Such observation highlights the crucial importance of reagent preparation and the need for rigorous quality control.

### 4.27. *Polygonum cuspidatum*

The root of *Polygonum cuspidatum* is usually formulated with several other ingredients and is most commonly prescribed for treating coughs, hepatitis, jaundice, amenorrhea, leucorrhea, arthralgia, burns, and snake bite [255]. A diversity of compounds have been isolated from *Polygonum cuspidatum*, dominated by resveratrol, polydatin, and anthraquinones and are presumed to be responsible for *Polygonum cuspidatum*'s anti-inflammatory, estrogenic, antitumour, antiaging, neuroprotective, and cardioprotective activities [256–258]. In one recent *in vivo* study examining wound healing in rats, extracts of *Polygonum cuspidatum* were found to increase TGF-*β*1 expression and to significantly improve wound healing in terms of reepithelization, granulation tissue formation, collagen synthesis, and angiogenesis [259]. Novel anthraquinones isolated from *Polygonum cuspidatum* have been verified to inhibit tyrosinase, the rate-limiting enzyme controlling the synthesis of melanin that gives colour to skin [260].

### 4.28. *Lithospermum erythrorhizon*

The dried root of *Lithospermum erythrorhizon* is indigenous to northeast China and has potent biological activities, including anti-inflammatory, antibacterial, antiangiogenic, and antitumour qualities [261]. Shikonin, a naphthoquinone, is extracted from the root of *Lithospermum erythrorhizon* and stimulates the activity of caspases, poly-(ADP-ribosyl) polymerase (PARP) and reactive oxygen species (ROS), triggering programmed cell death in cancer cell lines [262]. These characteristics prompted investigation of shikonin as a novel scar remediation therapy. These studies found that shikonin inhibits cell proliferation and collagen production in hypertrophic scar-derived human skin fibroblasts [263]. Arnebin-1, a related naphthoquinone extracted from *Lithospermum erythrorhizon*, has been reported to synergise with VEGF, resulting in significantly improved wound healing in a rat diabetic model [264].

### 4.29. *Rheum officinale*

*Rheum officinale*, also known as Chinese rhubarb, is one of the best known traditional herbal medicines with pharmacological activities. Extracts from the roots of *Rheum officinale* have strong antibacterial [265], antioxidative [266], anti-inflammatory [267], and haemostatic [268] effects, validating its widespread use for constipation, chronic liver and kidney diseases [265, 269], and skin lesions [270]. Using a rat excisional wound model, Tang et al. found healing was stimulated via TGF-*β*1-related pathways [270]. The nature of the active component responsible for this activity is not clear. Emodin [1,3,8-trihydroxy-6-methyl-anthraquinone], an anthraquinone derived from the roots of *Rheum officinale*, has been shown to act as a ligand for PPAR-*γ* and interact with HSP90 and androgen receptors, in part explaining its therapeutic benefit for chronic diseases [271]. Experimental evidence also indicates a direct association of emodin with NF-κB, AP-1, and STAT3, known regulators of proinflammatory cytokine and mitogenic kinase pathways [272, 273].

### 4.30. *Rhodiola imbricata*

*Rhodiola imbricata*, a perennial herb native to high altitudes (4000–5000 m) of the western Himalayas, is known to contain bioactive flavonoids, coumarins, and phenyl glycosides. These compounds are commonly found in botanical herbal medicines. Ethanolic extracts of rhizomes from *Rhodiola imbricata* stimulate a robust wound healing response when applied to excisional wounds in rats [274]. Others have reported related functions that may contribute to tissue repair, namely, immunomodulation [275], antioxidation [276], hepatoprotection [277], radioprotection [278], and anticancer [279] properties.

### 4.31. *Salvia miltiorrhiza*

The root of the perennial plant *Salvia miltiorrhiza* (also known as red sage) is highly valued in TCM and used to treat cerebrovascular and cardiovascular diseases, such as stroke, coronary heart disease, and hyperlipidemia [280–283]. To date, *Salvia miltiorrhiza* has been demonstrated to reduce ischemia and necrosis and to improve the survival of skin flaps after mastectomy [284, 285]. Salvianolic acids isolated from *Salvia miltiorrhiza* have potent antioxidative capabilities due to their polyphenolic structure [286]. Although hepatoprotective [287], neuroprotective [288], antimicrobial [289], anti-inflammatory [290], and anticancer [291] activities have been reported, the greatest clinical benefit of salvianolic acids appears to be cardiovascular protection, via the promotion of cardiac angiogenesis and inhibition of ischemia and hypoxia during myocardial injury [292]. Water-soluble extracts from *Salvia miltiorrhiza*, containing danshensu (DSU) and salvianolic acid B (SAB), have been shown to enhance the proliferation of fibroblasts and increase collagen synthesis [293]. Salvianolic acid B is also a potent antagonist of epithelial-to-mesenchymal transition, necessary for wound closure [294]. In contrast, cryptotanshinone, a lipid-soluble terpenoid isolated from *Salvia miltiorrhiza*, has been demonstrated to downregulate the expression of *COL1A1*, *COL3A1*, and *α-SMA* in hypertrophic scar-derived fibroblasts (HSF), as well as reduce HSF migration and HSF contraction, thus ameliorating fibrosis and scarring [295].

### 4.32. *Sanguisorba officinalis*

*Sanguisorba officinalis*, a member of the family Rosaceae and commonly known as great burnet, is widely distributed in the cooler northern districts of Asia, Europe, and North America [296]. Roots of this plant are a potent haemostatic [297], with antioxidant [298], immunomodulatory [298], anti-inflammatory [299], and antiallergy [300] properties. The traditional use of *Sanguisorba officinalis* is to control bleeding disorders. It is also applied to heal scalds, burns, allergic skin diseases, urticaria, eczema, and allergic dermatitis [299]. Aqueous extracts made from the root of *Sanguisorba officinalis* suppress mast cell degranulation, as well as inhibit activation of STAT-1, Jak-2, p38, and JNK pathways and release of inflammatory cytokines [301]. In mouse studies, the oral administration of polysaccharides isolated from *Sanguisorba officinalis* is claimed to stimulate wound contraction, reduce the time required for reepithelization (wound closure), increase collagen synthesis, and improve angiogenesis [296]. Administration of the polysaccharide extract also resulted in elevated IL-1*β* and VEGF in mice [296].

### 4.33. *Sophora flavescens*

*Sophora flavescens* is a species from a genus of over 50 plants distributed throughout Asia, Oceania, and the islands of the Pacific. The root of *Sophora flavescens* is used for conditions involving the heart, liver, intestinal tract, and skin. Experimental investigations indicate extracts from *Sophora flavescens* stimulate anticancer, antibacterial, antiviral, anti-inflammatory, and antipruritic responses and benefits wound healing [302]. One recent report claims it is a potent inhibitor of tyrosinase, the enzyme responsible for synthesizing melanin, thus has potential cosmetic applications as a skin whitener [303]. Other reports claim specific compounds present in *Sophora flavescens* benefit individuals with androgenetic alopecia [304]. Recently, Xu et al. demonstrated that a mixture of *Sophora flavescens* and other herbs significantly reduced perianal ulceration in a rat model, finding that the expression of prostaglandin E2 and IL-8 was concomitantly reduced in treated animals [302].

### 4.34. *Stemona tuberosa*

*Stemona tuberosa* is another of the 50 fundamental herbs used in TCM. It has strong insecticidal activity, the foundation property for its traditional use in treating impetigo, scabies, louse, lice, and ticks. It is also used as a mosquito repellent and preservative to protect stored cereals from insects [305]. In traditional medicine, it is used to treat coughs and lung infections. Alkaloid and stilbenoid isolated from the root of *Stemona tuberosa* are reported to have anti-inflammatory [306] and antibacterial [307] effects, while the dehydrotocopherol derivatives have been found to scavenge oxygen and free radicals [308]. Tocopherols isolated from the root of *Stemona tuberosa* increase cell proliferation in the mouse fibroblast NIH3T3 cells, suggesting the potential use of these compounds as wound healing agents [309].

### 4.35. *Wedelia trilobata*

The plant *Wedelia trilobata*, which is also known as *Sphagneticola trilobata*, was originally native to the tropical Americas; however, as one of the world's most invasive species, it is now ubiquitous throughout the tropics. Alcohol extracts made from the leaves of *Wedelia trilobata* have been used to treat rheumatism, stubborn wounds, and arthritic painful joints [310]. Luteolin, a flavonoid present in the leaves, has been demonstrated to contribute to the medicinal value of *Wedelia trilobata*, conferring neuroprotective, anticancer, antioxidant, and immunomodulatory activities [311]. Traditional healers use the leaves of *Wedelia trilobata* to treat skin wounds. Luteolin inhibits the expression of NF-κB-regulated proinflammatory cytokines, a characteristic feature of skin infection and psoriasis [312]. In a study designed to validate this traditional use, Balekar et al. fractionated ethanolic extracts from the leaves of *Wedelia trilobata* and assayed them *in vitro* [310]. Specific subfractions were found to support fibroblast viability, proliferation, and migration. Different subfractions were also found to be active against *Staphylococcus aureus* and *Staphylococcus epidermidis* [310].

### 4.36. *Zanthoxylum bungeanum*

*Zanthoxylum bungeanum* is a flowering plant belonging to the Rutaceae family, native to eastern provinces of China. It yields important food ingredients such as sichuan pepper [313]. Over 140 compounds have been isolated from *Zanthoxylum bungeanum*, including alkaloids, terpenoids, flavonoids, and free fatty acids, eliciting a wide variety of biological responses, including analgesic [314], anticancer [315], antioxidant [316], anti-inflammatory [317], antibacterial, antifungal, and antiasthma properties [318]. *Zanthoxylum bungeanum* are known in traditional Western folk medicine as “toothache trees,” useful for treating pruritus (itch) and chronic pain. The pericarp from the fruit berry is commonly used to formulate TCM oils, powders, tinctures, elixirs, and pills [319]. Extracts from *Zanthoxylum bungeanum* are also prescribed for skin infections, including acne, eczema, scalds, and wound healing [320]. One unique property of fruit husk extracts from *Zanthoxylum bungeanum* is as a lifting agent for skin wrinkles. When applied topically to skin, subcutaneous muscles are relaxed, reducing skin wrinkles, thus has attracted the attention of cosmetic manufacturers [321]. Another interesting property reportedly associated with essential oils of *Zanthoxylum bungeanum* is the capacity to enhance percutaneous drug delivery [322].

## 5. Conclusion

We have surveyed and presented an overview of evidence that explains why many medicinal plants are used as traditional treatments for cutaneous wounds and clinical skin disorders. Medicinal plants have been the first line of treatment for trauma, infection, disease, and injury from prehistory. Over millennia, humans have learned to identify and transform the botanical resources from the immediate environment, and with the development of trade, as food and medicine. A great many of these “ancient” and traditional medical plants have been validated to confer therapeutic benefits, albeit not always in controlled clinical trials. One unexpected outcome from validation studies is just how many medical plants synthesize equivalent or closely related compounds. Consequently, it is not surprising that many biological properties are also shared by unrelated species. Also shared are many of the same biological targets and pathways; many of these are also key events in the mammalian wound healing cascade. Many of the identified compounds target mitogenic pathways (e.g., AKT, PI3K, SMAD, and cyclins), the proinflammatory NF-κB pathway (e.g., caspases, interleukins, TNF-*α*, and TGF-*β*1), angiogenesis pathway (e.g., VEGF), extracellular matrix synthesis (e.g., MMPs), and differentiation pathways (e.g., *α*-SMA).

The active ingredients, part of use, type of extract, assessment methods, bioactivities, clinical use, formulation, and commercial product of the medicinal plants are summarized in Table 1. While experimental evidence has been acquired for each documented plant from *in vitro* or *in vivo* analyses, not every mechanism of action has been verified. On the contrary, several compounds, including acemannan (from *Aloe vera*), hydroxysafflor yellow A (from *Carthamus tinctorius*), polysaccharide (from *Ganoderma lucidum*), phthalide lactones, and alkaloids (from *Ligusticum striatum*), saponins (from *Panax ginseng*), shikonin and arnebin-1 (from *Lithospermum erythrorhizon*), salvianolic acids (from *Salvia miltiorrhiza*), polysaccharides (from *Sanguisorba officinalis*), and alkaloid and stilbenoid (from *Stemona tuberosa*) are well characterised and have been demonstrated to have properties that benefit wound healing. In particular, *Centella asiatica*, *Curcuma longa,* and *Paeonia suffruticosa* are popular medicinal products in several global markets.

We provide these data in the belief that we still have much to learn from traditional practices, some of which undoubtedly could deliver novel reagents and therapies for today's therapeutic challenges. Notwithstanding, we recognise that modern medicine and drugs remain effectively inaccessible (and unaffordable) to the majority of the world's population. For this reason alone, traditional medicine continues to be the first line of treatment, indeed, frequently the only line of treatment for many. With greater understanding of traditional practices comes appreciation and benefit to more of the world's peoples. We would like to see that this knowledge is not discarded by “modern medicine” but leveraged through investigation to benefit all.

## Figures and Tables

**Table 1:** Summary of the medicinal plants used in wound healing.

Botanical name	Traditional name	Plant family	Active ingredients	Part used	Type of extract	Assessment methods	Bioactivities	Clinical use	Formulation	Commercial product
*Aloe vera*	Lu Hui	Asphodelaceae	Acemannan [40]	Leaves	Ethanol	Punch biopsy wounds in Sprague-Dawley rats [40]	Immunomodulatory [40] Antiviral [40]	Wound healing [34]	Gel [323]	Aloe vera gel
*Arctium lappa*	Niu Bang Zi	Asteraceae	Arctigenin [324] Arctiin [42] Caffeic acid [42] Chlorogenic acid [42] Diarctigenin [42] Inulin [42] Lappaol F [49] Tannin [42] Trachelogenin 4 [42]	Leaves Whole root	No extraction, whole leaves Aqueous	Human burn wounds [53] Canine dermal fibroblast adhesion assay [52]	Anticancer [49] Antidiabetic [46] Anti-inflammatory [45] Antimicrobial [47] Antiviral [48] Hepatoprotective [50] Radical scavenging [44]	Acne vulgaris [43] Boils [42] Burns [53] Rashes [42] Sore throat [42] Wrinkles [51]	Ointment [53]	Not available
*Astragalus propinquus* *Rehmannia glutinosa*	Huang Qi Di Huang	Fabaceae Orobanchaceae	Astragaloside IV [57] Calycosin [57] Catalpol [57]	Roots	Aqueous	*In vitro* scratch wound healing and quantitative cell migration assays [59]	Anti-inflammatory [57] Proangiogenic [57]	Diabetic foot ulcer [56]	Herbal drink [56]	Not available
*Ampelopsis japonica*	Bai Lian	Vitaceae	Catechins [325] Epicatechin gallate [325] Resveratrol [325]	Root tuber	Ethanol	Cutaneous scald injury in rats [63]	Anticancer [62] Neuroprotective [61]	Antipyretic detoxicate [60] Burns [60] Ulcers [60]	Wound plaster (Patent: CN105748741A)	Hydrating Moisturizer
*Andrographis paniculata*	Chuan Xin Lian	Acanthaceae	Andrographolide [64] Kalmeghin [64]	Leaves	Aqueous	Excision model in albinos Wistar rats [76]	Anticancer [66] Antidiabetic [70] Anti-inflammatory [69] Antimalarial [73] Antimicrobial [71] Antioxidant [68] Antiviral [72] Hepatoprotective [74] Hypotensive [75] Immunostimulatory [66]	Dysentery [65] Fever [64] Snake bites [66] Sores [67]	Paste (applied externally), juice (internally) [326]	Chuan Xin Lian Nei Zhi Di Wan (穿心莲内酯滴丸)
*Angelica sinensis*	Dang Gui	Apiaceae	Ferulic acid [83] *n*-Butylidenephthalide [86]	Whole plant	Ethanol	Cell line antioxidant activity assay [83]	Anticancer [80] Anti-inflammatory [79] Antioxidant [81] Immunomodulatory [82]	Amenorrhea [78] Dysmenorrhea [78] Menstrual disorders [78]	Ointment [327] Nanosilver hydrocolloid dressing [328]	Dang Gui Shao Yao San (当归芍药散)
*Blumea balsamifera*	Ai Na Xiang	Asteraceae	L-Borneol [92]	Leaves	Violate oil	Excision wound model in mice [92]	Antifungal [90] Antiobesity [91] Antiplasmodial [88] Antitumour [89]	Beriberi [87] Dermatitis [87] Eczema [87] Skin bruises [87] Skin injury [87]	Oil	Blumea leaf oil
*Boswellia sacra*	Ru Xiang	Burseraceae	Boswellic acids [329]	Resin	Dry extract Dry extract	Rabbit ear hypertrophic scar model of full-thickness scar defect [96] Excision wound model in diabetic C57BL/6 mice [97]	Anticancer [329] Antifibrotic [97]	Improvement of blood circulation [95] Pain treatment [94] Rheumatoid arthritis [93]	Spray [93]	Frankincense oil
*Caesalpinia sappan*	Su Mu	Fabaceae	Brazilin [103] Sappanchalcone [103]	Roots Roots	Ethanolic Ethanol	*In vitro* antibacterial assay [102] *In vitro* anti-inflammatory and wound healing assays [103]	Antiallergic [99] Antibacterial [102] Anti-inflammatory [100] Viral neuraminidase inhibitory [101]	Improvement of blood circulation [98] Pain treatment [98] Oedema [98]	Tablets	Lukol™, Vicco Vajradanti™
*Calendula officinalis*	Jin Zhan Ju	Asteraceae	Esculetin [108] Quercetin-3-O-glucoside [108]	Flower Flower Flower	Hexane and ethanol Hydroethanol Hexane and ethanol	Scratch assay [107] Excision wound model in BALB/c mice [109] Punch wound model in rats [110]	Antibacterial [106] Anticancer [106] Antifungal [106] Anti-inflammatory [106] Antioxidant [106] Antiviral [106]	Burns [105] Dermatitis [105] Wounds [104]	Topical spray [330] Oil [331]	Calendula Herbal-Extract Toner Plenusdermax®
*Camellia sinensis*	Cha Shu	Theaceae	Epcatechin-3-gallate [111] Epicatechin [111] Epigallocatechin [111] Epigallocatechin-3-gallate [111]	Leaves Leaves Leaves	Methanol Methanol Ethanol	*In vitro* and *in vivo* keloid fibroblasts models [125] NIH3T3 fibroblast proliferation assay [115] Excision wound model in Sprague-Dawley rats [121]	Antiaging [116] Anticarcinogenic [115] Anti-inflammatory [113] Antimicrobial [114] Antiobesity [117, 118] Antioxidant [112] Cardioprotective [119] Neuroprotective [120]	Angina pectoris [332] Asthma [332] Bacterial infections [332] Cancer [332]	Oil	Tea tree oil
*Carthamus tinctorius*	Hong Hua	Asteraceae	Hydroxysafflor yellow A [130]	Seeds	Reflux	Antioxidant enzyme assay in zebrafish [333]	Anti-inflammatory [132] Antioxidant [131] Apoptosis-inhibiting [134] Melanogenesis-inhibitory [129] Proangiogenic [133]	Blood stasis [334, 335] Osteoporosis [334, 335] Promotion of bone formation [334, 335]	Oil	Safflower oil (红花油)
*Celosia argentea*	Qing Xiang	Amaranthaceae	Celosin I [139] Celosin II [139]	Leaves	Ethanol	Rat burn wound model [137]	Antidiabetic [140] Antimicrobial [141] Antioxidant [138] Hepatoprotective [139]	Skin sores [137] Ulcers [137]	Poultice of stems and leaves (topically) [336]	Not available
*Centella asiatica*	Ji Xue Cao	Apiaceae	Asiaticoside [144] Madecassoside [146]	Aerial parts	Hexane, ethyl acetate, methanol, and water	Incision and partial-thickness burn wound models in rats [142]	Anti-inflammatory [142] Antioxidant [142] Proangiogenic [142]	Wounds [142]	Oral form (tablets, drops) Topical medication (ointments and powder) Injections (subcutaneous and intramuscular) [337, 338]	Madecassol® [143] Centellase® Blastoestimulina® [337] Collaven® [338]
*Cinnamomum cassia*	Rou Gui	Lauraceae	Cinnamaldehyde [147]	Bark Whole plant	Volatile oil Ethanol	*In vitro* and *in vivo* angiogenic activity assay [147] Excision wound model in rats [339]	Anticancer [151, 152] Antidiabetic [340] Anti-inflammatory [150] Antimicrobial [341] Antioxidant [342]	Analgesia [147] Improvement of blood circulation [147]	Oil	Cinnamon cassia oil
*Commiphora myrrha*	Mo Yao	Burseraceae	Furanoeudesma-1,3-diene [165] Terpene [165]	Leaves and resin Resin Leaves and resin	Dry extract Dry extract Dry extract	Excision wound model in diabetic rats [164] Human recurrent aphthous stomatitis [165] *In vitro* cell migration assay [166]	Analgesic [159] Antibacterial [158] Anti-inflammatory [157] Antioxidant [156]	Gastrointestinal diseases [161, 162] Wounds and pain [163, 165]	Oil	Myrrh essential oil
*Curcuma longa*	Jiang Huang	Zingiberaceae	Curcuminoids [170]	Rhizomes	Nanosuspension	Antioxidant analysis [343]	Antibacterial [344] Anti-inflammatory [345] Antioxidant [346]	Digestive diseases [347] Liver disorders [347] Menstrual difficulties [347] Pain disorders [347] Sprains [347] Wounds [347]	Capsules [348]	Kordel's Theracurmin™ BCM-95®, Theracurmin™, CurcuVIVA™, CurcuMIND, Long-vida RD CAVACURMIN®, Biocurcumax™ [349]
*Daphne genkwa*	Yuan Hua	Thymelaeaceae	Daphnodorin B [177] Daphnodorin G [177] Daphnodorin G 3ʺ-methylether [177] Daphnodorin H [177] Daphnodorin H 3-methyl [177] Daphnodorin H 3ʺ-methylether [177] Genkwanin [177] Genkwanol A [177] Yuanhuacine [350] Yuenkanin [177]	Flower Roots	Aqueous	Human wounds from anal fistula therapy [171]	Anti-inflammatory [175] Antitumour [177] Immunoregulatory [178, 179] Melanogenesis inhibitory [172]	Coughs [174] Wounds [171]	Not available	Not available
*Entada phaseoloides*	Ke Teng	Fabaceae	Tannin [182]	Stem skin and seeds	Ethanol	Acetic acid-induced mouse writhing experiment [351]	Antibacterial [180] Antioxidant [181]	Aging [352] Atherosclerosis [352] Cancer [352] Diabetes [352] Neurodegenerative disorders [352]	Not available	Not available
*Hibiscus rosa-sinensis*	Zhu Jin	Malvaceae	Anthocyanins [353] Flavonoids [353] Polyphenolic acids [353] Protocatechuic acid [353]	Flower	Ethanol	Excision, incision and dead space wound models in rats [187, 188]	Antibacterial [186]	Antitumour [353] Hair growth [184]	Powder	Lustrous Henna® Shampoo powder
*Ganoderma lucidum*	Ling Zhi	Ganodermataceae	Ganoderma lucidum polysaccharide [204]	Fruiting body Fruiting body	Aqueous Aqueous	Excision wound model in diabetic rats [190] Full-thickness excision wound model in diabetic mice [204]	Antihyperlipidemic [197] Anti-infective [192–194] Anti-inflammatory [354] Antioxidant [195] Cardioprotective [196] Immunomodulating [191]	Cancer [198] Diabetes [200] Hepatitis [189] Leukaemia [189] Ulcer [201]	Not available	Ganoderma lucidum spores powder capsules
*Ligusticum striatum*	Chuan Xiong	Apiaceae	Ferulic acid [206] Ligustilide [206] Senkyunolide A [206] Tetramethylpyrazine [206]	Rhizome	Extracted by hydrodistillation	Hypertrophic scar rabbit model [208]	Antiatherosclerotic [355] Antioxidant [356] Neuroprotective [357] Vasorelaxant [358]	Headache [206] Ischemic disorders [205] Menstrual disorders [207]	Not available	Chuanxiong chatiao Wan (川芎茶调丸)
*Lonicera japonica*	Jin Yin Hua	Caprifoliaceae	Biflavonoids [209] Dicaffeoylquinic acid [209] Phenolic acids [209] Quercetin [209]	Flowering aerial parts	Ethanol	Rat excision wound model [212]	Anticancer [210] Antihyperlipidemic [210] Anti-inflammatory [211] Antimicrobial [211] Antioxidant [211] Hepatoprotective [210]	Infectious diseases [209]	Essential oils [210]	Not available
*Paeonia suffruticosa*	Mu Dan	Paeoniaceae	Suffruticosides A, B, C, and D [359] Galloyl-oxypaeoniflorin [359] Galloyl-paeoniflorin [359]	Root bark	Water, ethanol-water	*In vitro* cell viability and proliferation assays [220]	Antidiabetic [218] Anti-inflammatory [217] Antioxidant [214] Antitumour [216] Neuroprotective [215]	Genital diseases [360] Improvement of blood circulation [361]	Ointment	Winvivo (Puji) ointment
*Panax ginseng*	Ren Shen	Araliaceae	Ginsenosides Rb1, Rb2, Rc, and Rd [232]	Leaves Leaves, root, and whole plant	Ethanol Dichloromethane, ethanol, butanol, and methanol	Laser burn and excision wounds models in mice [236] Cell migration and wound healing assays [221,237–239]	Antiaging [230] Antiallergic [229] Anticancer [227] Anti-inflammatory [225] Antimicrobial [228] Antioxidant [226] Immunomodulating [231]	Wound healing [221]	Not available	Ginseng Strong 200 mg Ginsemax^®^ Ginseng Vita-Complex
*Panax notoginseng*	San Qi	Araliaceae	Notoginsenoside Ft1 [240]	Leaves, flower, roots, and rhizome	Steamed extraction	*In vitro* anticoagulation and antioxidation test; *in vivo* hemostasis and anti-inflammation test [362]	Angiogenesis-stimulatory [240, 241] Anticancer [246] Antidiabetes [247] Anti-inflammatory [243] Antioxidative [242] Immunostimulatory [244] Neuroprotective [245]	Trauma [240, 241]	Powder on wound Spray on wound	San Qi Fen (三七粉) Yunnan Baiyao (云南白药)
*Polygonum cuspidatum*	Hu Zhang	Polygonaceae	Emodin [260] Polydatin [258] Resveratrol [256]	Roots	Ethanol	Full-thickness excision wounds in rats [259]	Antiaging [256, 257] Antibacterial [260] Anticancer [256, 257] Anti-inflammatory [256, 257] Antioxidant [256, 257] Antiviral [260] Cardioprotective [256, 257]	Hepatitis [255] Hyperlipemia [255] Jaundice [255] Scald [255] Skin burns [255] Suppurative dermatitis [255]	Capsules	Resveratrol supplement
*Lithospermum erythrorhizon*	Zi Cao	Boraginaceae	Arnebin-1 [264] Shikonin [363]	Roots	Frozen and ground	Apoptotic effects against murine primary peritoneal macrophages [364]	Antiangiogenic [261] Antibacterial [261] Anti-inflammatory [261] Antiscarring [263] Antitumorigenic [261]	Wounds [264]	Oil formulations, gel [365]	Burt's bee Res-Q ointment (神奇紫草膏)
*Rheum officinale*	Da Huang	Polygonaceae	Emodin [270]	Roots	Ethanol	Rat excisional wound model [270]	Antibacterial [265] Anti-inflammatory [267] Antioxidative [266] Haemostatic [268]	Chronic kidney disease [269] Hepatitis [265] Wounds [270]	Pills	Dahuang Zhechong Wan (大黄蜇虫丸)
*Rhodiola imbricate*	Hong Jing Tian	Crassulaceae	Gallic acid [277] *p*-Tyrosol [277] Rosavin [277] Rosin [277]	Rhizome	Ethanol	Rat excision wound model [274]	Anticancer [279] Antioxidative [276] Hepatoprotective [277] Immunomodulatory [275] Radioprotective [278]	Asthma [277] Fatigue [277] Hemorrhage [277] Impotence [277] Gastrointestinal diseases [277]	Not available	Not available
*Salvia miltiorrhiza*	Dan Shen	Lamiaceae	Cryptotanshinone [366] Danshensu [293] Salvianolic acid B [293]	Leaves Leaves	Aqueous Aqueous	*In vitro* proliferative and angiogenic assays; second-degree burn wound model in rats [367] Cell proliferation assay; *in vitro* assays for collagen and melanin synthesis [293]	Anticancer [291] Anti-inflammatory [290] Antimicrobial [289] Antioxidant [286] Antiplatelet aggregation [368] Proangiogenic [367, 369, 370]	Blood stasis [371, 372] Cardiovascular diseases [282]	Pills	Compound danshen dripping pills (复方丹参滴丸)
*Sanguisorba officinalis*	Di Yu	Rosaceae	Tannins [296] Triterpenoid glycosides [296] Triterpenoids [296]	Roots	Ethanol	Burn wound model in mice [296]	Antiallergy [300] Anti-inflammatory [299] Antioxidant [298] Haemostatic [297] Immunomodulatory [298]	Burns [299] Chronic intestinal infections [373] Haemorrhoids [373] Menorrhagia [373] Scalds [299]	Oral administration	*Sanguisorba officinalis* Mother Tincture
*Sophora flavescens*	Ku Shen	Fabaceae	Kushenol [374] Sophoraflavanone B [374]	Roots	Cellulose column	Human liver LO2 proliferation and viability assay [375]	Analgesic [376] Anthelmintic [376] Antipyretic [376] Skin whitening [376] Stomachic [376]	Asthma [374] Burns [374] Dysentery [374] Eczema [374] Fever [374] Hematochezia [374] Inflammatory [374] Jaundice [374] Oliguria [374] Vulvar swelling [374]	Gel	Kushen gel (苦参凝胶) Gatuline® Spot-Light
*Stemona tuberosa*	Bai Bu	Stemonaceae	Tuberostemonine N [306]	Roots	Methanol	Mouse fibroblast NIH3T3 proliferation assay [309]	Antibacterial [307] Anti-inflammatory [306] Antioxidant [308] Antitussive [305] Neuroprotective [377]	Insect pests [307]	Not available	Not available
*Wedelia trilobata*		Asteraceae	Kaurenoic acid [310] Luteolin [311]	Leaves	Ethanol	*In vitro* antimicrobial assay, cell proliferation, and viability assays [310]	Antimicrobial [310] Antioxidant [311] Antitumour [311]	Arthritic painful joints [310] Rheumatism [310] Stubborn wounds [310]	Not available	Not available
*Zanthoxylum bungeanum*	Hua Jiao	Rutaceae	Afzelin [378] Hyperoside [378] Quercitrin [378] Rutin [378]	Seed oil	Press extraction	Rat scald wound model [378]	Anaesthetic [314] Antiasthma [318] Anti-inflammatory [317] Antoxidant [316] Antitumour [315]	Skin wrinkles [321]	Cream	ZANTHALENE®

## Abbreviations

a.k.a.: Also known as
AKT: Protein kinase B
ALT: Alanine aminotransferase
ANBP: *Agrimonia eupatoria* (A), *Nelumbo nucifera* (N), *Boswellia sacra* (B), and pollen from *Typha angustifoliae* (P)
AP-1: Activator protein 1 (transcription factor)
*α*-SMA: Alpha smooth muscle actin
AST: Aspartate aminotransferase
COX-2: Cyclooxygenase-2
CTGF: Connective tissue growth factor
DAMPs: Damage-associated molecular patterns
DSU: Danshensu
ECM: Extracellular matrix
EGCG: (-)-Epigallocatechin-3-gallate
ERK: Extracellular signal-regulated kinase
ET-1: Endothelin 1
HSF: Hypertrophic scar-derived fibroblasts
HSYA: Hydroxysafflor yellow A
IL-1*α*: Interleukin-1 alpha
IL-1*β*: Interleukin-1 beta
IL-6: Interleukin-6
IL-8: Interleukin-8 (CXCL8)
IL-10: Interleukin-10
iNOS: Inducible nitic oxide synthase
Jak-2: Janus kinase 2
JNK: c-Jun *N*-terminal kinase
MAPK: Mitogen-activated protein kinase
MEK: Mitogen-activated protein kinase, MAPK/ERK kinase
MMP-1: Matrix metalloproteinase-1 (interstitial collagenase)
MMP-2: Matrix metalloproteinase-2 (gelatinase A)
MMP-3: Matrix metalloproteinase-3 (stromelysin-1)
MMPs: Matrix metalloproteinases
mRNA: Messenger ribonucleic acid
mTO: Mammalian target of rapamycin
NF–κB: Nuclear factor-kappa B
PAMPs: Pathogen-associated molecular patterns
PARP: Poly-ADP-ribosyl polymerase
PGE2: Prostaglandin E2
PI3K: Phosphatidylinositol-4,5-bisphosphate 3-kinase
ROS: Reactive oxygen species
SAB: Salvianolic acid B
SBP: Shexiang Baoxin Pill
SMAD: Sapien homologue of mothers against decapentaplegic
STAT-1: Signal transducer and activator of transcription 1
STAT3: Signal transducer and activator of transcription 3
TCM: Traditional Chinese medicine
TGF-*β*1: Transforming growth factor-beta 1
TNF-*α*: Tumour necrosis factor-alpha
TXA_2_: Thromboxane A2
VEGF: Vascular endothelial growth factor
WHO: World Health Organisation
5-LOX: Arachidonate 5-lipoxygenase.
